# Exploring paediatric rheumatology care: a ten-year retrospective analysis of the patient population in Ghana

**DOI:** 10.1186/s12969-024-00975-3

**Published:** 2024-03-21

**Authors:** Dzifa Dey, Bright Katso, Afia Baah, Saudatu Isaaka, Emmanuella Amoako

**Affiliations:** 1 Rheumatology Unit, Department of Medicine and Therapeutics, University of Ghana Medical School Legon-Accra, Accra, Ghana; 2 Rheumatology Unit, Department of Medicine and Therapeutics, Korle Bu Teaching Hospital, Accra, Ghana; 3 Department of Medicine and Therapeutics, Korle Bu Teaching Hospital, Accra, Ghana; 4 Department of Paediatrics, Korle Bu Teaching Hospital, Accra, Ghana

**Keywords:** Paediatric rheumatic disease, Africa, Rheumatology, Disease characteristics, Teaching hospital

## Abstract

**Background:**

Rheumatic diseases can seriously impact children’s general health, development, and growth. However, due to a lack of resources, paediatric rheumatology is a largely underdeveloped speciality in many African nations. Children with rheumatic disorders face obstacles in accessing specialized medical care, including lack of specialists, care centres, medication access, and limited research and education to increase understanding of paediatric rheumatic disease among healthcare practitioners. This study described the disease characteristics, prevalence, and challenges faced by paediatric rheumatic disease patients receiving care at a teaching hospital in Accra, Ghana.

**Methods:**

A retrospective record-based study was conducted among all paediatric cases presenting to the rheumatology clinic of the Korle Bu Teaching Hospital (KBTH) from January 2011 to December 2021. Data collected include clinical features, laboratory findings at disease presentation, andtherapeutic regimens prescribed per standard guidelines and experiences.

**Results:**

A total of 121 cases were identified as of 2021, indicating a point prevalence of 0.0011%. The majority (73%) were females with a mean age of 13.4 ± 3.2 years. The mean duration of symptoms in months experienced by patients before being successfully referred to a rheumatologist was 18 months. There were significant differences between referred and confirmed diagnoses, especially in cases involving mixed connective tissue diseases (MCTD), systemic lupus erythematosus (SLE), and juvenile dermatomyositis (JDM), suggesting that these conditions may be under-recognised. Arthralgia and arthritis were the most common presenting symptoms. More than three-quarters (86.8%) of the cases studied were treated with steroids (oral or intravenous). In cases requiring immunosuppressive therapy, methotrexate was the most commonly prescribed in 33.9% of instances. Mortality was recorded at 8.3%, with the majority involving SLE cases. Most (95.7%) of the primary caregivers expressed positive experiences regarding care received at the adult rheumatology clinic.

**Conclusion:**

There were significant delays in diagnosis and diagnostic accuracy for patients with paediatric rheumatic disease (PRD). This highlights the pressing need for strengthening paediatric rheumatology services in Africa, including increasing awareness about these conditions among the public and healthcare providers to improve early diagnosis and quality of life for children with these conditions.

**Supplementary Information:**

The online version contains supplementary material available at 10.1186/s12969-024-00975-3.

## Background

Rheumatic diseases are a broad category of chronic diseases characterised by persistent inflammation, usually with unknown aetiology, that affects structures of the musculoskeletal system, blood vessels, and various tissues [1]. Worldwide, these conditions are a leading cause of disability, significantly impacting the economies and social systems of countries [2, 3]. Children and adolescents are also affected by rheumatological conditions known as paediatric rheumatic disease (PRD). Globally, 6–7 million children are estimated to have rheumatological conditions, with over 78% of them residing in Asia and Africa [4–6].

Paediatric rheumatic disease (PRD) poses a significant risk of mortality and long-term disability if they are not promptly identified and treated effectively [7]. Juvenile idiopathic arthritis (JIA) is one of the most prevalent paediatric rheumatic disease and exhibits a significant burden across various aspects of the patients’ lives. A study involving 310 children diagnosed with JIA for a minimum of 5 years revealed that around 20% of them experienced moderate to severe disability. This included being limited to little or no personal duties as usual, and being largely or wholly bedridden or confined to a wheelchair, with little or no ability to perform self-care. Additionally, 10% exhibited major impairment in Health-Related Quality of Life (HRQOL), 33.2% had joint damage, and 25% showed extra-articular damage [8]. In another study of patients diagnosed with systemic-onset juvenile idiopathic arthritis (SOJIA), damage-related disability was present in 43% of the patients [9]. According to a retrospective record analysis, 13% of the 1,081 JIA patients studied had uveitis, with complications occurring in 37% of cases, where 15% of the patients with uveitis have had to do 62 eye operations [10]. Individuals with JIA were also found to significantly exhibit a higher occurrence of psychiatric problems (35%) compared to their age and sex-matched controls (12.5%) [11]. Using arthritis and other rheumatic conditions (AORC) codes, the Center for Disease Control and Prevention’s (CDC) National Center for Health Statistics (NCHS) death data indicated that approximately 1,000 children under 15 years old died due to complications from arthritis and other rheumatic conditions between 1979 and 1998, averaging 50 deaths per year [12]. These risks associated with PRD become more severe when the diagnosis is delayed. Additionally, such delays result in psycho-social effects during adolescence [13].

In low-middle-income countries, epidemiological data on PRD is severely limited [14]. Estimates of the prevalence of PRD in such countries are difficult to ascertain. The situation is considerably more challenging in Sub-Saharan Africa (SSA), where paediatric rheumatology services are not readily available, and healthcare providers often exhibit lower levels of awareness and management of paediatric rheumatic diseases [15, 16]. Given these healthcare hurdles, children living with rheumatic conditions in SSA are more likely to have severe disease, high morbidity and mortality, poorer quality of life, and worse mental health outcomes [17, 18].

The need for research to deliver sustainable solutions for paediatric rheumatic disease (PRD) patients in the low-middle income countries of SSA is imperative. Improving the knowledge base of the different paediatric rheumatic diseases in this region is of utmost importance, particularly in medical schools and residency training programs, to increase awareness among specialists and general practitioners for early referral and diagnosis [14, 16].

The current study was conducted to explore paediatric rheumatology care in Ghana. Little is known in the field of PRD in this country. Spanning a decade from 2011 to 2021, it represents the most extensive study period compared to prior research in West and East Africa [19–22]. The study aims to examine care at the adult rheumatology clinic and shed light on the nature of this understudied sub-speciality towards mitigating long-term morbidity and mortality resulting from delayed referral and diagnosis.

## Methods

This was a retrospective record-based study involving paediatric rheumatic disease (PRD) seen over ten years, from January 2011 to December 2021. The study was conducted at the rheumatology unit of the Korle Bu Teaching Hospital (KBTH) in Accra, Ghana. The KBTH is Ghana’s premier tertiary healthcare institution, with a capacity of approximately 2000 beds and 21 clinical and diagnostic departments providing healthcare to the public. Furthermore, KBTH serves as the national referral centre in Ghana and ranks as the third-largest referral centre in Africa. Most of its patient population comes from the southern regions of Ghana, and it also extends its services to neighbouring countries, including Burkina Faso, Nigeria, Togo, and Benin. Ghana has no pediatric rheumatology clinic. Instead, paediatric rheumatic disease patients receive care from adult rheumatologists in collaboration with paediatricians at the hospital. Patients displaying signs and symptoms suggestive of rheumatic diseases may be seen as referrals through the rheumatology unit’s Out-Patient Department (OPD) or emergencies through the hospital’s Paediatric Emergency Department (PED). These referrals usually originate from peripheral healthcare facilities nationwide.

Inclusion criteria for this study were paediatric cases referred to the rheumatology unit at KBTH from January 2011 to December 2021 and diagnosed with PRD based on validated diagnostic criteria outlined by the International League of Associations for Rheumatology (ILAR), the American College of Rheumatology (ACR), the European Alliance of Associations for Rheumatology (EULAR), and other established criteria for rheumatic diseases [23–26]. Exclusion criteria included individuals over 18 years of age at disease presentation, those with inconclusive rheumatic disease diagnoses, patients with incomplete medical records, and individuals diagnosed with a rheumatic disease outside the defined study period.

The rheumatology unit at KBTH maintains a database of patients with rheumatic diseases. Currently, the Clinical Rheumatology Unit in the Department of Medicine renders outpatient services to > 1,700 patients. On average, 40 follow-up patients attend weekly clinics with at least 6 new cases each week. Two adult rheumatologists run the service, supported by two residents in training and rotating internal medicine residents. Available support services include general physiotherapist services and a dedicated psychologist. Relevant data for the participants in this study were obtained by accessing their medical records from this database. A structured data extraction sheet was employed for data collection. Information was retrieved from patients’ medical records, encompassing a range of data points, including demographics, clinical and laboratory characteristics at the time of diagnosis, medical treatment, and outcomes. Specifically, the collected variables comprised; age at disease presentation to the rheumatology clinic, sex, duration of the disease from diagnosis at the rheumatology clinic to study, comorbid conditions, signs and symptoms at presentation to the rheumatology clinic, referral diagnosis, referring facility, final diagnosis, duration of initial symptoms before specialist consultation, diagnostic investigations performed, details of the standard of care, and disease outcomes. Baseline and diagnostic tests done included complete blood count (CBC), erythrocyte sedimentation rate (ESR), C-reactive protein (CRP), Liver function tests and antinuclear antibody (ANA) via enzyme-linked immunosorbent assay ELISA technique, Hepatitis B and C (due to its endemic nature for treatment decisions as the standard of care), Extractable nuclear antigen ENA, Rheumatoid factor and Anti Cyclic Citrulinated Peptide (anti CCP). Serological tests were conducted at only one time point due to financial constraints.

The study also included a cross-sectional aspect where data was collected from the primary caregivers of patients meeting the inclusion criteria to ascertain challenges to paediatric patient care at the adult clinic. A questionnaire was designed with an informed consent statement, and all caregivers who participated provided consent.

The data was analysed using Statistical Package for the Social Sciences (SPSS) version 26.0. Frequencies and percentages were the descriptive statistics used to describe all categorical variables and continuous variables were presented using means and standard deviations. To ascertain the agreement between primary and final diagnoses in the study, referral letters with details of the primary diagnosis from general practitioners were compared with the final diagnosis made by rheumatologists. Discrepancies or consistencies were noted, and descriptive statistics were used to report findings. The prevalence was calculated as point prevalence, using the total population of children 18 years or younger in Ghana as of 2021 and the total confirmed PRD cases as of 2021.

The study was approved by the University of Ghana Medical School’s Department of Community Health Review Committee with approval number UGMS-CHDRC-028/2022.

## Results

**Fig. 1:**
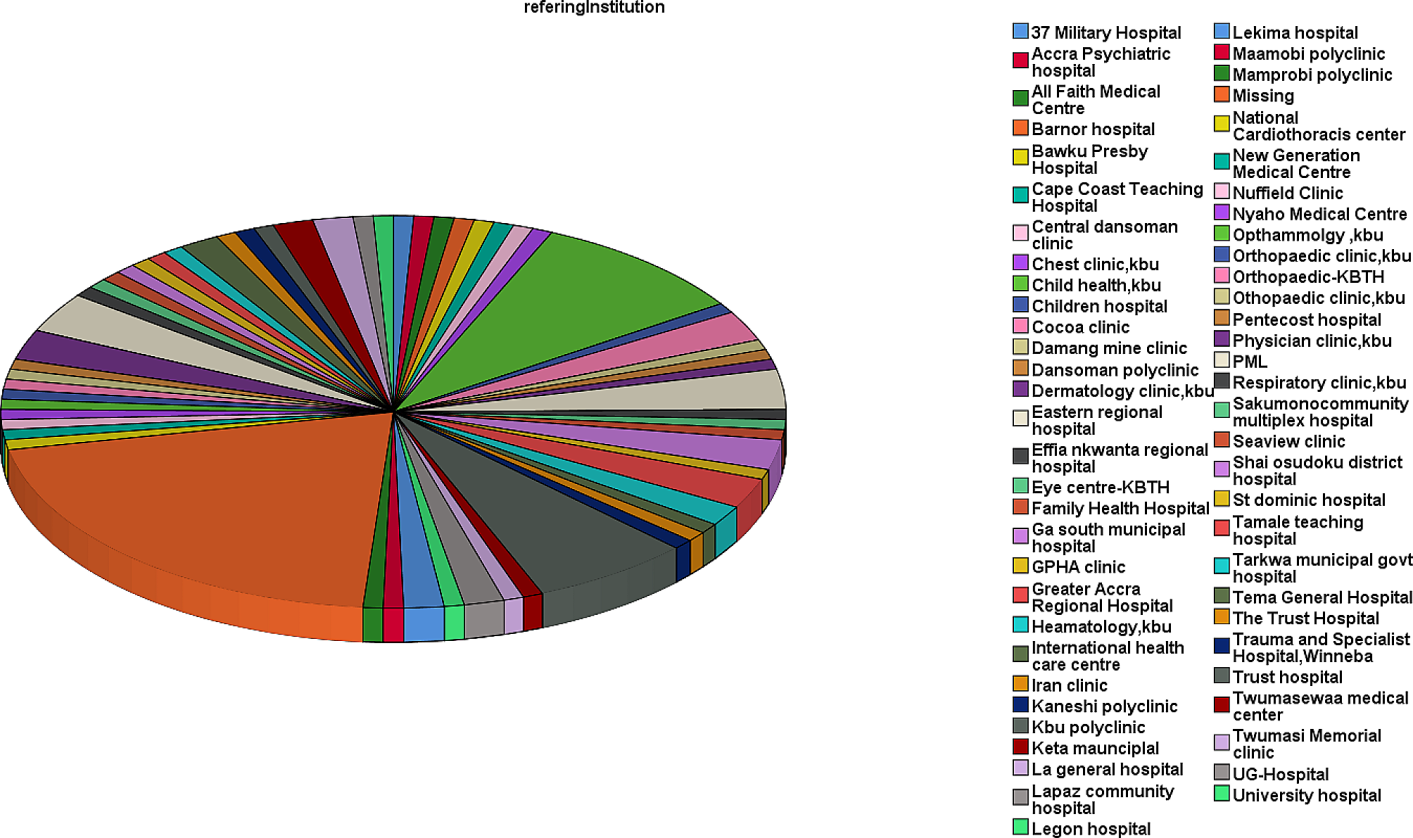
Pie chart showing different facilities referring patients to the rheumatology clinic

This study reviewed and analysed medical records of one hundred and twenty-one (121) PRD patients at the rheumatology unit of KBTH, all of whom met the inclusion criteria. This accounted for 6.0% of the total 2,013 rheumatological cases the unit saw over the study period. The PRD cases consisted of 89 (73.6%) females and 32 (26.4%) males, resulting in a female-to-male ratio of approximately 3:1. The mean ± SD age of the patients was 13.4 ± 3.2 years, with the minimum and maximum ages of 3 and 18 years. The mean disease duration from diagnosis to the study was 4.5 ± 2.8 years (range: 1–10 years). The mean duration of the onset of first signs and symptoms before consulting a rheumatologist was 18 ± 17.9 months. Most patients (94.2%) had no underlying medical conditions. However, 3.3% had sickle cell disease, 1.7% were asthmatic, and 0.8% had peptic ulcer. These PRD cases were referred from various healthcare facilities across the country. The majority (61.2%) were from governmental health facilities, including municipal, district, regional, and teaching hospitals in Ghana, as illustrated in Fig. 1.

The most common signs and symptoms at disease presentation were joint pains and swelling (100%), fever (45.5%), rash (41.3%), weight loss (40.5%), fatigue (34.7%), and oral ulcers (19.8%). Results for the hepatitis test show 1 (0.8%) reactive/positive case for hepatitis B and no reactive/positive case for hepatitis C. (see Table 1).

**Table 1:** Demographic and clinical features at disease presentation

Characteristic	*n* = 121	%
Age, (mean ± SD years) (13.4 ± 3.2)		
Duration of symptom (mean ± SD months) (18.0 ± 17.9)		
Duration of disease, (mean ± SD years) (4.5 ± 2.8)		
Sex		
* Female*	89	73.6
* Male*	32	26.4
Comorbidity		
* Sickle cell disease*	4	3.3
* Asthma*	2	1.7
* Peptic ulcer*	1	0.8
* None*	114	94.2
Hepatitis		
* B - tested Positive*	731	60.3
* C– tested Positive*	710	58.7
Signs and symptoms		
* Fatigue*	42	34.7
* Fever*	55	45.5
* Anorexia*	21	17.4
* Weight loss*	49	40.5
* Morning stiffness*	19	15.7
* Rash*	50	41.3
* Hair loss*	21	17.4
* Oral ulcers*	24	19.8
* Photosensitivity*	15	12.4
* Eye pain*	10	8.2
* Joint pains and joint swelling*	121	100.00
* Abdominal pain*	16	13.2
* Chest pain*	19	15.7
* Cough*	17	14.0
* Oedema*	14	11.6
* Muscle weakness*	20	16.5
* Paralysis*	6	5.0

From Table 2, juvenile idiopathic arthritis (JIA) was the most common PRD case, accounting for 48.8% of the study’s total cases. This was followed by systemic lupus erythematosus (SLE) in 34.7% of the cases and mixed connective tissue disease (MCTD) in 8.3%. The remaining were juvenile dermatomyositis (JDM) 3.3%, polymyositis (1.7%), vasculitis (0.8%), Takayasu’s (0.8%), Marfan’s syndrome (0.8%) and fibromyalgia (0.8%). Comparisons between referral diagnoses and final diagnoses revealed some differences in the primary and final diagnosis match for various conditions. For juvenile idiopathic arthritis (JIA), 64% (38/59) of cases showed a match between primary and final diagnoses. For systemic lupus erythematosus (SLE), only 43% (18/42) had a match. Mixed connective tissue disease (MCTD) and juvenile dermatomyositis (JDM) exhibited lower match rates, with only 10% (2/10) and 25% (1/4) respectively. Conversely, there were no primary-final diagnosis matches for Vasculitis, Takayasu’s syndrome, and Fibromyalgia. Notably, cases of Polymyositis and Marfan’s syndrome demonstrated a 100% match between primary and final diagnoses.

**Table 2:** Referring diagnoses vs. final diagnoses confirmed by a rheumatologist

Referring diagnoses			Vs	Confirmed diagnoses		
	n	%			n	%
JIA	38	31.4		JIA	59	48.8
SLE	18	14.9		SLE	42	34.7
MCTD	2	1.7		MCTD	10	8.3
JDM	1	0.8		JDM	4	3.3
Polymyositis	2	1.7		Polymyositis	2	1.7
Vasculitis	0	0.0		Vasculitis	1	0.8
Takayasu’s	0	0.0		Takayasu’s	1	0.8
Marfan’s syndrome	1	0.8		Marfan’s syndrome	1	0.8
Fibromyalgia	0	0.0		Fibromyalgia	1	0.8
Non-specific rheumatological diagnoses, e.g., chronic kidney disease, dermatitis, recurrent multiple joint pain, muscular dystrophy, etc.	59	48.7		Not confirmed	0	0.0
**Total**	**121**	**100**		**Total**	**121**	**100**

Table 3 shows the routine laboratory tests for confirming diagnosis among the study cases, including complete blood count (CBC), erythrocyte sedimentation rate (ESR), c-reactive protein (CRP), and liver function test (LFT). At the time of this study, 56 (46.2%) patients had no record of an antinuclear antibodies (ANA) test. Of the remaining 65 (53.8%) with ANA records, 61.5% (40/65) had positive results, while 38.5% (25/65) had negative results. Records for 21 patients were identified for extractable nuclear antigens (ENA), with results showing 28.6% (6/21) positive and 71.4% (15/21) negative. Only 54.2% (32/59) of the JIA patients had records for anti-cyclic citrullinated peptide (Anti-CCP) antibodies, and results ranged from 0.05 to 2267 units per millilitre (u/ml). Also, 97% (57/59) of JIA patients who had records of rheumatoid factor (RF) showed 24.6% (14/57) positive and 75.4% (43/57) negative. Among the SLE, the complements 3 (C3) distribution ranges from 0.69 to 182 milligrams per deciliter (mg/dL), while the complements 4 (C4) ranges from 0.10 to 43 mg/dL.

**Table 3:** Routine laboratory results

Parameter	Mean	Median	Std. deviation	Minimum	Maximum	Normal range
Hemoglobin (HB)	10.810	10.800	1.9335	4.8	15.5	11.5–15.5 g/dL
White Blood Cells (WBC)	8.525	7.705	4.2085	1.0	22.7	4.5–13.5 × 10^3/µL
Platelets (PLT)	369.96	339.00	154.675	65	984	150–450 × 10^3/µL
Erythrocyte Sedimentation Rate (ESR)	53.88	37.00	44.165	2	150	0–20 mm/h
C-Reactive Protein (CRP)	34.791	8.385	67.7941	0.1	420.0	< 10 mg/L
Serum Albumin (S-ALB)	41.94	39.00	44.702	4	464	35–50 g/L
Complement 3	55.79	39.50	55.545	1	182	90 to 180 mg/dL,
Complement 4	10.35	6.00	11.772	0	43	10 to 40 mg/dL
Anti-CCP	97.49	2.80	400.959	0	2267	> 20 U/mL

Table 4 lists the standard of care medications among the study cases. Steroids (oral and intravenous) and proton pump inhibitor (PPI) drugs were the predominant treatments recorded in the study and given to 105 (86.8%) of the patients. Calcium supplementation was also provided to approximately 105 (86.8%) patients, whereas 64 (52.9%) and 15 (12.4%) cases were treated with hydroxychloroquine and nonsteroidal anti-inflammatory drugs (NSAIDs), respectively. Immunosuppressive agents used include methotrexate, azathioprine, mycophenolate mofetil, and cyclophosphamide in 33.9% (41/121), 20.6% (25/121), 11.6% (14/121), and 10.7% (13/121) cases respectively. No biologics were recorded as treatment options in any of the cases studied.

**Table 4:** Patients’ medications

Medications	Total = 121	JIA	SLE	MCTD	JDM	Polymy -ositis	Vascu- litis	Takaya- su’s	Marfan’s syndrome	Fibro-myalgia
Hydroxychloroquine	64	19(29.7)	35(54.7)	6(9.4)	3(4.6)	1(1.6)	0(0.0)	0(0.0)	0(0.0)	0(0.0)
Prednisolone (oral)	100	46(46.0)	39(39.0)	9(9.0)	2(2.0)	2(2.0)	1(1.0)	0(0.0)	0(0.0)	1(1.0)
IV Methylprednisolone	5	1(20.0)	1(20.0	1(20.0	1(20.0	0(0.0)	0(0.0)	1(20.0)	0(0.0)	0(0.0)
Omeprazole	105	47(44.8)	40(38.1)	10(9.5)	3(2.9)	2(1.9)	1(0.9)	1(0.9)	0(0.0)	1(0.9)
Calcium	105	47(44.8)	40(38.1)	10(9.5)	3(2.9)	2(1.9)	1(0.9)	1(0.9)	0(0.0)	1(0.9)
Mycophenolate mofetil	14	0(0.0)	12(85.7)	2(14.3)	0(0.0)	0(0.0)	0(0.0)	0(0.0)	0(0.0)	0(0.0)
Azathioprine	25	2(8.0)	18(72.0)	4(16.0)	1(4.0)	0(0.0)	0(0.0)	0(0.0)	0(0.0)	0(0.0)
Methotrexate	41	33(80.5)	4(9.8)	3(7.3)	1(2.4)	0(0.0)	0(0.0)	0(0.0)	0(0.0)	0(0.0)
IV Cyclophosphamide	13	2(15.4)	9(69.2)	1(7.7)	0(0.0)	0(0.0)	1(7.7)	0(0.0)	0(0.0)	0(0.0)
Folic acid	40	32(80.0)	3(7.5)	4(10.0)	1(2.5)	0(0.0)	0(0.0)	0(0.0)	0(0.0)	0(0.0)
NSAIDs	15	7(46.6)	4(26.6)	0(0.0)	0(0.0)	0(0.0)	1(6.7)	1(6.7)	1(6.7)	1(6.7)

During the study period, there were 10 fatalities recorded, constituting 8.3% of the total cases reviewed. The majority of these mortalities (50%) were SLE cases, followed by MCTD (30%) and JIA (10%).

Only 70 of the primary caregivers could be successfully reached to provide their consent and complete the questionnaire. The majority, 67 (95.8%) of these caregivers were parents, 48 (68.7%) were mothers, and 19 (27.1%) were fathers. All the caregivers visited an average of 2.3 ± 0.27 different facilities before being referred to the adult rheumatology clinic at KBTH. Only 23 (32.9%) had an experience with a paediatric clinic before coming to the adult rheumatology clinic at KBTH. A significant majority, 67 (95.7%) of the caregivers, indicated they had limited knowledge of their child’s condition. However, the majority (95.7%) indicated they had positive experiences with waiting time (95.7%), treatment given (100%), and relationships with caregivers (98.6%) at the adult rheumatology clinic. The majority (68.6%) also had no challenges with the treatment given to their child. Nearly all the caregivers, specifically 92.9% are satisfied with the notion of their children continuing to receive care at the adult clinic. Factors reported by these caregivers as having driven their satisfaction include the perceived quality of care, the child’s stabilization, the welcoming atmosphere, and the supportive nature of the healthcare professionals present at the adult rheumatology clinic in KBTH. Furthermore, the majority (74.3%) were very comfortable with their children being among adults at the clinic. (see Table 5).

**Table 5:** Experiences and challenges of caregivers at the adult rheumatology clinic

	*n* = 70	%
Type of caregiver		
* Mother*	48	68.7
* Father*	19	27.1
* Other (e.g., sister, brother, grandparent)*	3	4.2
Number of facilities visited.		
* 1*	21	30.0
* 2*	16	22.9
* 3*	21	30.0
* Above 3*	11	17.1
Knowledge about a child’s diagnosis		
* No*	67	95.7
Preference for clinic		
* Adult*	65	92.9
Experiences		
* Waiting time– positive*	118	95.7
* Treatment given– positive*	121	100.0
* Relationship of care provider– positive*	120	98.6
Challenges		
* No Challenge*	48	68.6
* Tablet too Big*	1	1.4
* Tablet too Many*	1	1.4
* Drugs are Expensive*	12	17.1
* Bitter Drugs*	7	10.0
* Prefer Syrup*	1	1.4

## Discussion

Compared to earlier studies [19, 20], this is the most extended study period on PRD in West Africa and one of the longest in Africa. The female predominance is easily noticed in our study, with nearly three-quarters (73.6%) of the total cases reviewed being females, which is comparable to other studies on PRD in the sub-Saharan African region [19, 21, 27]. However, in other low-middle-income countries, especially Asia, studies show the female-to-male ratio to be almost equal (1:0.9) [14], while others show slight male predominance [28]. This suggests potential regional variations, particularly between Africa and Asia in the sex distribution of PRD patients, highlighting the need for further research to explore underlying factors contributing to these differences.

The mean age in years at disease presentation to the rheumatology clinic reported by our study was 13.4 ± 3.2 years. This is similar to earlier studies in other parts of West Africa, which recorded 14 years [19] and 12.7 years [20]. These are comparatively higher than observed in other studies in the southern and eastern parts of Africa and Asia, where ages below 10 years were recorded, lower than the figures reported in West Africa [14, 21, 27, 28]. These variations in age at disease presentation between West Africa and other regions like southern and eastern Africa and Asia could imply differences in disease prevalence and subtype, health-seeking behaviour, healthcare access and awareness among populations in these regions warranting further investigation for better understanding.

On average it took 18 months, a minimum of one month, and a maximum of 108 months for patients to be referred to a rheumatologist following symptoms onset in our study. This closely matches the findings of a related study in Nigeria, which also reported a mean duration of symptom onset-to-diagnosis of 18.4 months [19]. This long duration of symptom onset-to-diagnosis could be attributed to factors such as low PRD awareness, lack of accessibility to specialised healthcare, and the absence of paediatric rheumatology clinics in Ghana. The cases studied were referred from various health facilities nationwide, with the majority (61.2%) from government health facilities such as district and municipal hospitals. All caregivers visited an average of 2.3 ± 0.27 different facilities before their referral to the adult rheumatology clinic at Korle Bu. Thus, the rheumatologist was at least the third clinician to see the patient, which further emphasises the earlier position that there is a lack of initial recognition or low awareness and delayed access to specialised care for PRD in Ghana.

The current study reported gaps in the provisional diagnosis from primary care referrals and final diagnoses by the rheumatologist. Accurate matching between referral and final diagnoses was seen in 64% of JIA cases, 43% in SLE, 10% in MCTD, and 25% in JDM. The confirmed SLE, MCTD, and JDM diagnoses are significantly higher than the referring diagnoses, suggesting that these conditions may be under-recognised among general physicians in Ghana. The levels reported in this study are lower than the 84.6% reported by Migowa et al. in Kenya [22] but considerably higher than the 18.8% match reported in Asia [14]. Generally, the issue of primary-final diagnoses match is present not only in less developed countries but also in developed countries. Nevertheless, it is more common in low-middle income countries [29], partly attributed to a lack of awareness in these parts of the world [30]. Increasing the awareness of rheumatic disorders among general practitioners and the public is likely to enhance the diagnostic agreement in Sub-Saharan Africa and promote optimal health outcomes in children affected by the disease.

Arthralgia and arthritis were the most common disease manifestations reported by all cases (100%). Earlier studies on PRD in other parts of Sub-Saharan Africa similarly reported a high prevalence of this manifestation, 91.2% by Olaosebikan et al. [19] and 84.6% by Adelowo et al. [21]. Systemic complaints were also common, with 45.5% having a fever, close to the study in Yemen by Dahman, which showed 51.4% of participants having a fever. Our study reported JIA as the most frequently confirmed diagnosis, comprising 48.8% of all cases, followed by SLE in 34.7% and MCTD in 8.3%. Furia et al. reported the same order of occurrence in their study of PRD in Tanzania. Both studies are similar to reports of PRD studies in other parts of sub-Saharan Africa that reported JIA predominance in the study population [16, 19] as well as other regions outside Africa [31, 32].

Past research underscored the significance of laboratory tests in diagnosing and monitoring PRD. These tests serve various purposes, such as confirming suspected diagnoses, assessing disease severity, and tracking treatment effectiveness [33]. While only a select few tests are part of the diagnostic criteria, the most effective approach for screening patients for PRD involves a comprehensive clinical assessment, including a thorough patient history and a detailed physical examination, especially targeting the musculoskeletal exam [34, 35]. The rheumatology clinic at KBTH follows this approach to ensure positive outcomes for patients, regardless of their caregivers’ financial circumstances. In Ghana, like many other African countries, basic diagnostic tests such as complete blood count (CBC), rheumatoid factor (RF) and anti-nuclear antibodies (ANA) are available, however, these are limited to the capital cities [36, 37]. Additionally, there is poor funding of rheumatology health services by the state as seen across the African continent [38, 39], such that PRD patients in Ghana are compelled to pay out of pocket, resulting in the greater majority of patients not being able to afford to pay for these simple investigations. In this study, baseline laboratory investigations were requested for all patients, including complete blood count (CBC), erythrocyte sedimentation rate (ESR), C-reactive protein (CRP), Liver function tests and antinuclear antibody (ANA). However, due to financial constraints, not all patients could undergo this preliminary work-up. Of the total 121 cases, only 63 could take the ANA test, representing 52%. Disease-specific tests such as ENA panel, Anti-CCP, and Complements 3 and 4 were ordered based on patients’ presentation as a more targeted approach and to reduce the financial burden on caregivers. Finding innovative ways to expand access to essential laboratory testing and healthcare resources, such as collaboration with healthcare providers, policymakers, and stakeholders, may be necessary to develop strategies for improving the diagnosis and management of PRD in Ghana and, for that matter, Africa.

The average haemoglobin levels for males and females at disease presentation were 11 g/dl and 10.7 g/dl, respectively, typically normocytic normochromic anaemia suggestive of anaemia of chronic disease. This suggests that both the male and female paediatric patients were anaemic at diagnosis. Average values recorded for ESR and CRP were 53.88 mm/hr and 34.79 mg/l, respectively, at disease presentation which is high for both sexes and suggestive of very active disease at presentation among the PRD cases.

The mainstay of treatment for PRD in this study includes steroids, omeprazole, calcium and hydroxychloroquine. These medicines are generally more affordable and accessible in sub-Saharan Africa except hydroxychloroquine [19, 21]. A number of the cases were treated with disease-modifying anti-rheumatic drugs (DMARDs), with methotrexate (80.5%) being the most commonly prescribed for the cases involving JIA while azathioprine (72.0%) and mycophenolate mofetil (85.7%) were commonly prescribed for cases involving SLE and MCTD. None of the PRD patients received biologics at the clinic. Biologics are expensive, and even when cost is not an issue, the necessary biologics to treat certain PRD may not be available in the country. This is a problem that has also been observed in the subregion, where the availability and affordability of biologic drugs such as rituximab and etanercept were significant concerns for paying clients who have to pay out of pocket and with limited health insurance coverage [19].

As opposed to past research that looked at paediatric rheumatology care in sub-Saharan Africa, the current study is unique in that it also examined challenges specific to the primary caregivers regarding care received at the adult clinic setting. Notably, countries like Ghana, Nigeria, and Tanzania in the sub-region do not have dedicated paediatric rheumatology care centres; hence, paediatric patients are primarily seen in adult rheumatology clinics [19]. We explored whether the healthcare experience at the adult clinic was satisfactory for a younger population from the experiences of caregivers. It was discovered that 95.8% of the primary caregivers were direct parents of the child, while the rest were relatives of the patients. This indicates that all the primary caregivers encountered at the clinic were family caregivers who had significant emotional bonds with the patient and were part of the patient’s family life circle. It is well-documented that familial caregivers contribute substantially to enhancing patients’ quality of life, whether within the hospital or home setting and play a pivotal role in assisting patients in coping with their illnesses [40].

The vast majority of the caregivers reported positive experiences regarding waiting times and their relationships with healthcare providers at the adult rheumatology clinic. They reported experiencing no difficulties with the treatment received, although some expressed concerns about the high cost of medications. Additionally, almost all of them were satisfied with their children receiving care at the adult rheumatology clinic. It is worth noting that, all caregivers visited an average of two different facilities before seeking care at the adult rheumatology clinic. This suggests that their favourable encounters and satisfaction with the adult rheumatology clinic at the KBTH were informed by their prior experiences across multiple healthcare settings including paediatric clinics. Thus, despite challenges in obtaining resources to establish paediatric rheumatology centres in sub-Saharan Africa, this speciality can still succeed if well-coordinated with adult rheumatology.

The study also revealed that a substantial majority (95.7%) of the caregivers demonstrated insufficient awareness of their child’s diagnosis. Therefore, comprehensive health education initiatives should be developed to increase awareness and knowledge among parents and caregivers of individuals living with PRD in Ghana. Improving the education of healthcare professionals and the general public, through conventional and digital media, regarding the key symptoms of PRD, particularly musculoskeletal symptoms, is crucial for early detection and prompt intervention towards improving diagnosis and patient outcomes. Additionally, establishing more streamlined referral systems, particularly in rural regions, is crucial to reducing the time between PRD symptom onset and consultation with a rheumatologist to mitigate financial constraints experienced by many caregivers. It is important to consider including DMARDs in insurance schemes, like the National Health Insurance in Ghana, to lessen the financial constraints experienced by many caregivers. Above all, encouraging extensive paediatric rheumatology research and education in Sub-Saharan Africa is critical due to significant knowledge gaps and would play a crucial role in enhancing awareness and facilitating early diagnosis and appropriate treatment, thus improving patient outcomes.

## Conclusion

This comprehensive study of Paediatric Rheumatic Disease (PRD) in West Africa reveals a female and JIA predominance and a delayed diagnosis-to-referral period. Discrepancies between provisional and final diagnoses underscore the need for increased awareness among healthcare providers. Musculoskeletal symptoms are the most common, and in the absence of other diagnoses, combined with constitutional symptoms such as fever may indicate PRD. The financial constraints experienced by caregivers to conduct appropriate laboratory investigations, coupled with the unavailability of DMARDs, especially biologics, may impact the standard of care for PRD. The positive experiences of the family caregivers highlight the success of a coordinated approach between adult and paediatric rheumatology in the absence of dedicated paediatric centres.

### Electronic supplementary material

Below is the link to the electronic supplementary material.

Supplementary Material 1

## Data Availability

The data supporting this study’s findings are available from the corresponding author upon reasonable request.

## Abbreviations

ACR: American College of Rheumatology
ANA: Antinuclear Antibody
Anti-CCP: Anti-Cyclic Citrullinated Peptide
CBC: Complete Blood Count
CRP: C-Reactive Protein
C3: Complement 3
C4: Complement 4
DMARDs: Disease Modifying Anti-Rheumatic Drugs
ENA: Extractable Nuclear Antigens
ESR: Erythrocyte Sedimentation Rate
g/dL: Grams per decilitre
g/L: Grams per litre
ILAR: International League of Associations for Rheumatology
JDM: Juvenile dermatomyositis
JIA: Juvenile idiopathic arthritis
KBTH: Korle Bu Teaching Hospital
MCTD: Mixed connective tissue disease
Mm/hr: Millimetres per hour
Mg/L: Milligrams per litre
NSAID: Nonsteroidal Anti-Inflammatory Drug
OPD: Out-Patient Department
PER: Paediatric Emergency Department
PPI: Proton Pump Inhibitor
PRD: Paediatric Rheumatic Disease
S-ALB: Serum Albumin
SLE: Systemic Lupus Erythematosus
SPSS: Statistical Package for Social Science
SSA: Sub-Saharan Africa
UGMS: University of Ghana Medical School
u/Ml: Units per Millilitre
