# Supplementary material for: Exploring paediatric rheumatology care: a ten-year retrospective analysis of the patient population in Ghana
Source: Pediatr Rheumatol Online J. 2024 Mar 21;22:40. doi: 10.1186/s12969-024-00975-3 (PMC10956341; doi:10.1186/s12969-024-00975-3)
Supplement: Supplementary file 1 — Supplementary Material 1 [file 12969_2024_975_MOESM1_ESM.docx]

**QUESTIONNAIRE**

**Title:** Exploring Paediatric Rheumatology Care: A Ten-Year Retrospective Analysis of The Patient Population in Ghana

**Investigator:** DZIFA DEY, Rheumatology Unit, Department of Medicine and Therapeutics, University of Ghana Medical School Legon-Accra, Ghana. +233244672343. [dzifakay@gmail.com](mailto:dzifakay@gmail.com)

Patient/Caregiver ID………………………

This study aims to investigate the experiences of primary caregivers of individuals aged 18 years or younger attending the adult rheumatology clinic at Korle Bu. Insights gained from this research can enhance awareness of the burden of pediatric rheumatologic conditions in Ghana, improving care and disease outcomes. As a participant, I acknowledge that my involvement is voluntary, and I may withdraw at any time without repercussions for me or the child. I understand that all information provided will be kept confidential and accessible only to the research team. My identity and that of my ward will remain anonymous and will not be associated with the research findings.

By signing below, I consent to participate in this study.

Date Caregiver’s signature

………………………… ……………………………

Please read through and give answers to the following questions.

1. Relation of caregiver to the patient

[ ] mother. [ ] father. [ ] other…………………………….

1. Do you feel you know enough about your child’s diagnosis?

[ ] yes. [ ] no

1. How many facilities have you visited for treatment before coming to the adult rheumatology clinic at Korle Bu?

[ ] one. [ ] two. [ ] three [ ] above three

1. Did you visit a paediatric clinic before coming to the adult rheumatology clinic?

[ ] yes. [ ] no

1. If no why……………………………………………………………
2. If yes, do you still get reviewed at paediatric clinic?

[ ] yes. [ ] no

1. a. Would you prefer to be seen there or at the adult rheumatology clinic at Korle Bu?

[ ] paediatric clinic. [ ] adult rheumatology clinic.

b. Please provide reasons for your answer………………………………………………………………………………………………………………………………………

1. What is your experience at the adult rheumatology clinic regarding;

*Waiting time?*

[ ] excellent. [ ] good. [ ] neutral. [ ] poor. [ ] very poor.

*Treatment given?*

[ ] excellent. [ ] good. [ ] neutral. [ ] poor. [ ] very poor.

*Relationship of care providers?*

[ ] excellent. [ ] good. [ ] neutral. [ ] poor. [ ] very poor.

1. Are you comfortable with your child among adult patients

[ ] yes. [ ] no

1. What challenge(s) have you faced following care received at the rheumatology clinic?

………………………………………………………………………………………………………………………………………………………………………………………………
